# Genetically distant bacteriophages select for unique genomic changes in *Enterococcus faecalis*

**DOI:** 10.1002/mbo3.1273

**Published:** 2022-03-16

**Authors:** Cydney N. Johnson, Dennise Palacios Araya, Viviane Schink, Moutusee Islam, Mihnea R. Mangalea, Emily K. Decurtis, Tuong‐Vi C. Ngo, Kelli L. Palmer, Breck A. Duerkop

**Affiliations:** ^1^ Department of Immunology and Microbiology University of Colorado School of Medicine Aurora Colorado USA; ^2^ Department of Biological Sciences University of Texas at Dallas Richardson Texas USA

**Keywords:** bacteriophages, coevolution, comparative genomics, *Enterococcus faecalis*

## Abstract

The human microbiota harbors diverse bacterial and bacteriophage (phage) communities. Bacteria evolve to overcome phage infection, thereby driving phage evolution to counter bacterial resistance. Understanding how phages select for genetic alterations in medically relevant bacteria is important as phages become established biologics for the treatment of multidrug‐resistant (MDR) bacterial infections. Before phages can be widely used as standalone or combination antibacterial therapies, we must obtain a deep understanding of the molecular mechanisms of phage infection and how host bacteria alter their genomes to become resistant. We performed coevolution experiments using a single *Enterococcus faecalis* strain and two distantly related phages to determine how phage pressure impacts the evolution of the *E. faecalis* genome. Whole‐genome sequencing of *E. faecalis* following continuous exposure to these two phages revealed mutations previously demonstrated to be essential for phage infection. We also identified mutations in genes previously unreported to be associated with phage infection in *E. faecalis*. Intriguingly, there was only one shared mutation in the *E. faecalis* genome that was selected by both phages tested, demonstrating that infection by two genetically distinct phages selects for diverse variants. This knowledge serves as the basis for the continued study of *E. faecalis* genome evolution during phage infection and can be used to inform the design of future therapeutics, such as phage cocktails, intended to target MDR *E. faecalis*.

## Graphical abstract

Characterizing the genetic mechanisms of bacteriophage‐bacteria interactions is critical for understanding how bacteria respond to and resist phage infection, and how phages evolve to counter such selection. This is especially relevant to the development of bacteriophage therapy against multidrug‐resistant bacterial infections. This study utilizes comparative genomic analyses to demonstrate how a pathogenic strain of Enterococcus faecalis responds to infection by two distantly related bacteriophages.

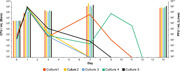

## 1 INTRODUCTION

*Enterococcus faecalis* is a Gram‐positive bacterium naturally residing as a commensal in the gastrointestinal tracts of animals, including humans (Lebreton et al., 2014). Immune suppression and/or antibiotic treatment promotes *E. faecalis* to outgrow and become a dominant member of the microbiota, leading to opportunistic infections (Ubeda et al., 2010). Strains of *E. faecalis* and *Enterococcus faecium* have acquired traits that allow them to survive host and environmental stresses, contributing to their success as pathogens (Lebreton et al., 2017; Tyne & Gilmore, 2014). The overuse of antibiotics in both medical and agricultural settings supports enterococcal pathogenesis by driving multidrug‐resistant (MDR) phenotypes (Gawryszewska et al., 2017; Munoz‐Price et al., 2005). As MDR *E. faecalis* infections continue to persist worldwide, there is a need to find alternative therapeutics capable of bypassing existing modes of antibiotic resistance (Esmail et al., 2019; Farman et al., 2019; Remschmidt et al., 2018).

Bacterial viruses, bacteriophages (phages), exist in high numbers in the intestinal tract where they infect and sometimes kill host bacteria, likely influencing the structure of the microbiota (Mangalea et al., 2021; Minot et al., 2013; Wandro et al., 2019). Due to their narrow host specificity and ability to lyse bacteria, phages are becoming an essential resource for the treatment of MDR bacterial infections (Kortright et al., 2019). Phage therapy offers many advantages over traditional antibiotics. For example, specificity can be tailored to target only the desired bacteria, leaving native microbes largely unaffected (Brives & Pourraz, 2020; Sulakvelidze et al., 2001). Additionally, phage replication is restricted to the abundance of the host, thus upon host exhaustion phages are depleted from the population (Moelling et al., 2018). In contrast, conventional antibiotics lack specificity, killing resident bacteria, and the compounds can remain in the patient after the infection has cleared (Langdon et al., 2016). There is an ever‐growing repertoire of phages that infect *E. faecalis* (Cook et al., 2021), making these promising candidates for phage therapy.

The development of successful phage therapies will require a complete understanding of the genetic interactions between phages and bacteria. Although phage therapy holds promise for the treatment of *E. faecalis* infections (Gelman et al., 2018; Khalifa et al., 2015), the molecular mechanisms of enterococcal phage infection and the bacterial host response to phage infection are understudied. Phage tail protein–receptor interactions underpin the molecular basis for phage strain specificity of the bacterial cell surface (Kabanova et al., 2019; Tu et al., 2017). To date, only the transmembrane protein PIP_EF_ (phage infection protein of *E. faecalis*) has been identified as a bona fide enterococcal phage receptor (Duerkop et al., 2016). Both phages VPE25 and VFW bind to *E. faecalis* by engaging with cell surface polysaccharides, and infection proceeds following viral DNA entry which requires PIP_EF_ (Duerkop et al., 2016). Studies in *E. faecium* have identified cell wall polysaccharides, secreted antigen A, and RNA polymerase to be involved in phage infection (Canfield et al., 2021; Wandro et al., 2019). Other studies have identified the enterococcal polysaccharide antigen (Epa) as a coreceptor for *E. faecalis* phages (Chatterjee et al., 2019; Ho et al., 2018).

Bacteria implement various mechanisms, including CRISPR‐Cas and restriction‐modification systems, to resist phage infection (Dupuis et al., 2013). However, spontaneous mutation is the main mechanism driving both phage resistance and phage‐bacteria coevolution (Oechslin, 2018). These spontaneous mutations are often located in genes encoding macromolecules found in the bacterial cell surface that prevents phage binding (Bishop‐Lilly et al., 2012; Denes et al., 2015; Eugster et al., 2015; Seed et al., 2012; Tu et al., 2017). To persist in the population, phages must mutate to counter host mutations; phage genomes are plastic, allowing for the accumulation of adaptive mutations (Burmeister et al., 2021; Koskella & Brockhurst, 2014). Although multiple studies evaluate bacterial mutations, and some evaluate phage mutations (Kupczok et al., 2018; Labrie & Moineau, 2007), during long‐term coevolution with pathogens posing a threat to human health (Denes et al., 2015; Eugster et al., 2015; Peters et al., 2020; Takeuchi et al., 2016), studies in clinically relevant enterococcal pathogens are limited. A recent experiment coevolving *E. faecium* and myophage EfV‐phi1 showed that phage tail fiber mutations helped overcome *E. faecium* phage resistance (Wandro et al., 2019).

To further our understanding of phage‐enterococcal interactions and their impact on genome evolution, we cocultured *E. faecalis* SF28073, an MDR strain resistant to vancomycin, gentamicin, and erythromycin, with two genetically distant phages, VPE25 and phage 47 (phi47) (Chatterjee et al., 2019; Duerkop et al., 2016). Both phages are long noncontractile‐tailed siphophages with double‐stranded DNA genomes and rely on the enterococcal polysaccharide antigen (Epa) for infection (Chatterjee et al., 2019, 2020). Epa of SF28073 has only been characterized based on predicted gene annotations (Palmer et al., 2012), and the biochemical structure has not been determined. The biochemical structure of Epa from a different MDR *E. faecalis* strain V583 has been determined (Guerardel et al., 2020). VPE25 has been confirmed to be a lytic phage (Duerkop et al., 2016), and we hypothesize that phi47 is lytic due to its lack of integrases, yet direct experiments need to be performed. Although both phages infect *E. faecalis* strain SF28073, nucleotide alignment revealed that their genomes only share 37.3% nucleotide identity, indicating they are genetically distinct. Orthologous protein clustering confirmed that these phages belong to unique enterococcal phage lineages. Based on these observations, we hypothesized that *E. faecalis* SF28073 may gain single‐nucleotide polymorphisms (SNPs) in genes involved in cellular pathways and macromolecule production that are specific to infection by either phage. To test this hypothesis, we ran two parallel coculturing experiments. In this report, predation by phages VPE25 and phi47 selects for unique genetic subpopulations of *E. faecalis* SF28073. We identified mutations in known macromolecule‐encoding genes previously demonstrated to be necessary for *E. faecalis* phage infection; however, numerous novel mutations were also identified within a lower percentage of the *E. faecalis* population. Our work shows that surface‐associated factors are the major driver of *E. faecalis* phage resistance, yet genetic alterations emerge that implicate diverse metabolic pathways in the *E. faecalis* response to phage infection. Additionally, our data suggest that the ratio of phage to bacteria is an important factor when studying phage–bacterial coevolution in vitro, as bottlenecks during serial passage may favor phage extinction.

## 2 MATERIALS AND METHODS

### 2.1 Routine bacterial culture

*E. faecalis* SF28073 (isolated from urine in 2003) (Oprea et al., 2004) was cultured in brain heart infusion (BHI, BD) medium at 37°C. Individual phi47‐resistant colonies were identified by inoculating washed bacterial samples from whole cultures into BHI and grown overnight at 37°C, normalizing overnight cultures to an optical density (OD) of 0.1, and serial dilutions were spotted onto THB agar containing 10 mM MgSO_4_ and 1 × 10^7^ PFU/ml of phi47. Plates were incubated overnight at 37°C and resistant isolates were picked, grown overnight in BHI at 37°C, and DNA was extracted and sequenced following the methods described below.

### 2.2 Phage isolation and quantification

Bacteriophages VPE25 (Duerkop et al., 2016) and phage 47 (phi47) (Chatterjee et al., 2019) were propagated using *E. faecalis* strains V583 (VPE25) or SF28073 (phi47) and phage titers were quantified by double agar overlay plaque assays, as described previously (Chatterjee et al., 2019; Duerkop et al., 2016). For clonal phage isolation, plaques were removed from agar overlays using a sterile p1000 pipette tip or a glass Pasteur pipette. Agar plugs were suspended in 1 ml sterile SM‐plus buffer (100 mM NaCl, 50 mM Tris‐HCl, 8 mM MgSO_4_, 5 mM CaCl_2_ [pH 7.4]) and eluted overnight at 4°C. The eluted phages were filtered through a 0.45 μm syringe filter and stored at 4°C before phage titer determination by plaque assay.

### 2.3 Coevolution assay

Individual colonies of *E. faecalis* SF28073 were grown overnight. The next day, 10^8^ colony‐forming units (CFU) of bacteria were inoculated into individual 125 ml flasks containing 25 ml of BHI broth supplemented with 10 mM MgSO_4_. Five flasks were infected with 10^5^ plaque‐forming units (PFU) of phage VPE25 and five flasks with 10^5^ PFU of phi47, originating from individual plaques. Bacteria‐only control cultures were included to identify mutations that arise due to laboratory passage in the absence of phage. All flasks were incubated at 37°C with shaking at 250 rpm. Every 24 h, the cultures were passaged by transferring 250 μl of the culture to flasks containing 25 ml of fresh BHI media supplemented with 10 mM MgSO_4_. At the time of passage, culture aliquots were removed for population DNA extraction and cryopreservation. For phi47, the culture media was centrifuged and filtered to isolate phages.

### 2.4 Testing bacterial cross‐resistance

From glycerol stocks of whole culture samples (bacteria and phage), BHI was inoculated, and the culture was grown overnight at 37°C with shaking at 250 rpm. Overnight cultures were washed three times with SM‐plus buffer to remove any extracellular phage. Bacterial pellets were resuspended in SM‐plus buffer and normalized to an OD of 0.1. Ten‐fold serial dilutions were spotted on THB agar plates containing 10 mM MgSO4 and 1 × 10^8^ PFU/ml of VPE25 or phi47. Bacteria coevolved with phi47 were spotted on VPE25 plates while bacteria coevolved with VPE25 were spotted on phi47 plates. CFU/ml was enumerated after overnight incubation at 37°C. Relative viability was calculated by dividing the CFU/mL of bacteria grown on phage agar plates divided by CFU/ml of bacteria grown in the absence of phage.

### 2.5 DNA extraction for population sequencing

Genomic DNA was isolated from 1 ml culture aliquots from coevolution cultures consisting of phage and bacteria using a previously described protocol for *E. faecalis* (Manson et al., 2003). Briefly, samples were treated with 5 mg/ml lysozyme for 30 min at 37°C. In all, 0.5% SDS, 20 mM EDTA, and 50 μg/ml Proteinase K were added and incubated at 56°C for 1 h. Samples were cooled to room temperature before adding an equal volume of phenol/chloroform/isoamyl alcohol and extracted by shaking. Samples were centrifuged at 17,000 rcf for 1 min, and the aqueous layer was extracted with an equal volume of chloroform. Again, samples were centrifuged at 17,000 rcf for 1 min, and nucleic acids were precipitated from the aqueous layer by adding 0.3 M NaOAc [pH 7] and an equal volume of isopropanol. Nucleic acid was pelleted by centrifuging at 17,000 rcf for 30 min at 4°C, washed with 70% ethanol, and centrifuged at 17,000 rcf for 10 min. Finally, the pellet was dried and resuspended in sterile water. Genomic DNA was sequenced using the Illumina NextSeq. 2000 platform to 300 Mbp depth at the Microbial Genome Sequencing Center (MiGS).

### 2.6 Hybrid assembly of the *E. faecalis* SF28073 genome

The *E. faecalis* SF28703 genome was sequenced using Oxford Nanopore Technology (ONT) as described previously (Jain et al., 2016; Wick et al., 2017). Briefly, 1.5 μg genomic DNA was mechanically sheared into 8 kb fragments with a Covaris g‐tube per the manufacturer's instructions before library preparation with the ONT Ligation Sequencing Kit 1D (SQK‐LSK108). Libraries were base called with MinKNOW (v3.5.5) to generate FASTQ and fast5 sequence reads. Illumina reads were obtained from MiGS as described above. Programs for DNA sequencing read processing and read assembly were run using the operating system Ubuntu 18.04.4 LTS. FASTQ sequences were filtered to gather reads with *q* scores >9 and length >1000 bp using Nanofilt (v2.5.0) (De Coster et al., 2018). The adaptor sequences were trimmed from the filtered reads with Porechop (v0.2.3) (https://github.com/rrwick/Porechop). The processed MinION reads were coassembled with Illumina reads using Unicycler (v0.4.7) with the default setting “normal mode” (Wick et al., 2017). Incomplete assemblies were manually completed as described in the “Unicycler tips for finishing genome” page (https://github.com/rrwick/Unicycler/wiki/Tips-for-finishing-genomes). Briefly, Bandage (v0.8.1) was used to visualize the completion status of the assembly (Wick et al., 2015), and unassembled contig sequences were extracted. Using these unassembled contig sequences as baits, long reads from MinION sequences were gathered for incomplete regions using minimap2 (v2.11‐r797) and an in‐Bandage BLAST search was performed with the long reads against the graph (Li, 2018). If long reads supported the continuity of two unassembled contigs, then the Bandage graph editing function was used to duplicate, delete edges, and merge contigs. The complete assembly sequence was saved from Bandage in FASTA format.

### 2.7 Analysis of serially passaged bacterial populations using Illumina sequencing

Illumina reads from the bacterial populations obtained from MiGS were mapped to the assembled *E. faecalis* SF28073 chromosome (GenBank accession number CP060804) and the three endogenous plasmids (pSF1, CP060801; pSF2, CP060802; and pSF3, CP060803) using CLC Genomics Workbench (Qiagen) with default settings. Detailed read mapping statistics were generated using the “QC for read mapping” tool in CLC Genomics Workbench with default settings to obtain the range of coverage and zero coverage regions in each assembly. The “Find low coverage” tool in CLC Genomics Workbench with the low coverage threshold set at 0 was used to manually inspect the regions found by the quality analysis to contain regions with 0 coverage. Sequence variants were identified using the “Basic variant detection” tool with a minimum coverage of 100, the minimum frequency of 30% (count/coverage] × 100), and a ploidy of 0. All variants identified were manually examined, and mutations not altering the amino acid sequence (silent) were excluded from the analysis. Variants present in the bacteria‐only controls were also excluded from further analysis.

### 2.8 Phage 47 genome sequencing and analysis

Phi47 genomic DNA was isolated following the methods described above. The phi47 genome was assembled de novo from Illumina reads using CLC Genomics Workbench following the default parameters, with the largest contig forming the full genome. Genome annotation was performed using RAST and NCBI BLASTp (v2.0) (Altschul et al., 1990; Aziz et al., 2008). During coevolution with *E. faecalis* SF28073, culture media was filtered through a 0.45‐μm filter. DNA was extracted from filtered media using the proteinase K and phenol/chloroform method described above and sequenced using Illumina technology at MiGS. These reads were mapped to the wild‐type phi47 genome and SNPs were identified using CLC Genomic Workbench with a minimum coverage of 15, a minimum frequency of 30%, and a ploidy of 0.

### 2.9 OrthoMCL analysis

Enterococcal phage phylogeny was determined using OrthoMCL (Li et al., 2003) as described previously (Canfield et al., 2021). Enterococcal phage genomes were downloaded from the INPHARED phage genome database (Cook et al., 2021). As of July 1, 2021, there were 126 enterococcal phage genome sequences available in addition to our inclusion of the phi47 genome. Proteomes determined using Prodigal (Hyatt et al., 2010) were used as input into an OrthoMCL MySQL database. A cluster inflation value of 1.5 was used and the resulting matrix was input for ggdendro and ggplot2 packages in R version 3.6.3. The dendrogram was determined using the average linkage method for hierarchical clustering of Manhattan distance metrics.

## 3 RESULTS

### 3.1 Phi47 and VPE25 phages are genetically distinct

**Figure 1:**
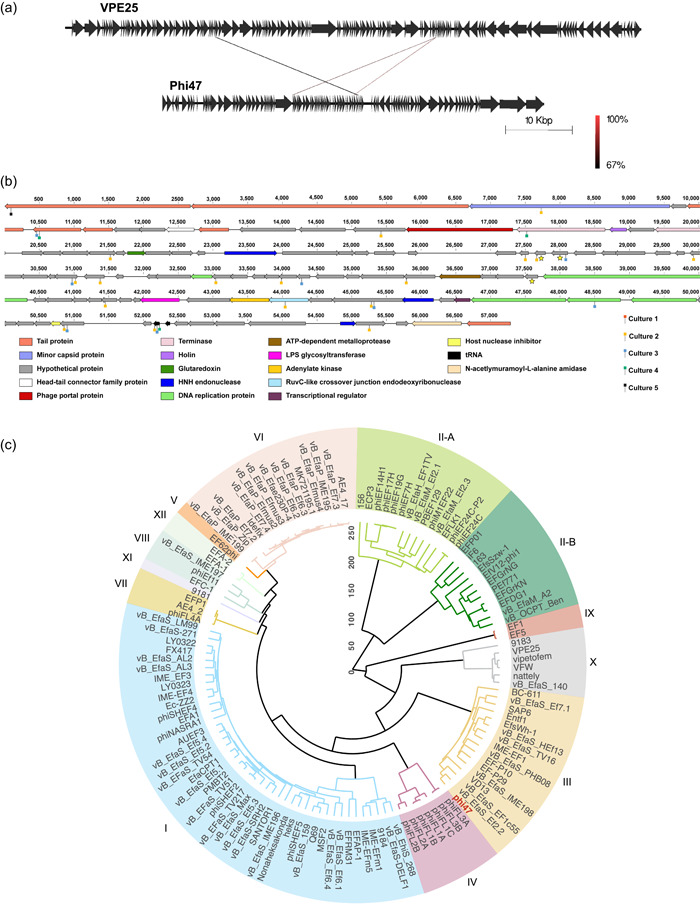
Phi47 and phage VPE25 are genetically distinct. (a) EasyFig analysis shows three genes shared between phage 47 and VPE25. These genes are 67%–100% identical at the nucleotide level. (b) Genome annotation predicted the function of 29 open reading frames and 2 tRNAs. Open reading frames encoding proteins of similar functions are depicted in the same color. A similar figure for phage VPE25 can be found in the 2016 manuscript by Duerkop et al. Stars indicate the genes shared in Figure 1a. Lollipops represent genes with identified nonsynonymous mutations found in each culture. Lollipop color corresponds with culture number. (c) OrthoMCL was used to compare the phage 47 genome to all publicly available enterococcal phage genomes. A phylogenetic tree was generated from OrthoMCL. Height is the average linkage of hierarchical clustering with 1000 iterations using the Manhattan distance metric. In all, 126 enterococcal phage genomes from the INPHARED database were used for comparison to phi47 (in red text). Distinct phage orthoclusters are represented by colored boxes. Roman numerals next to shaded boxes designate the orthocluster number

Phi47 and VPE25 are both siphoviruses belonging to the class Caudoviricetes (Chatterjee et al., 2019; Duerkop et al., 2016; Turner, Kropinski, et al., 2021). However, these phages differ in host range and genome content. VPE25 is a virulent phage capable of infecting numerous *E. faecalis* strains (Duerkop et al., 2016), including SF28073, while phi47 primarily infects SF28073 (Chatterjee et al., 2019). Phi47 depends on the enterococcal polysaccharide antigen (Epa) for adsorption to host cells (Chatterjee et al., 2019). EasyFig comparison of the phage genomes revealed only three shared genes (lines, Figure 1a) (Sullivan et al., 2011). These genes exhibit 67% or greater identity at the nucleotide level.

The phi47 genome is 57,289 base pairs in length, consisting of 101 predicted open reading frames (ORFs) and two tRNAs. Using RAST genome annotation (Aziz et al., 2008) and the NCBI BLASTp platform, we characterized the phi47 genome based on functional classifications (Figure 1b). The genome exhibits typical modularity; meaning that tail, structural, and DNA replication genes are in proximity to genes of similar function. The remainder, and the majority of the genes, are predicted to be hypothetical. The genome organization for VPE25 has been previously published (Duerkop et al., 2016).

Comparative genomic analysis of phi47 was performed with all publicly available enterococcal phage genomes using OrthoMCL (Figure 1c) (Cook et al., 2021). This algorithm generates a phylogenetic tree of clustered phage genomes (orthoclusters) based on orthologous proteins (Bolocan et al., 2019; Canfield et al., 2021; Li et al., 2003). Of the 11 known orthoclusters (Bolocan et al., 2019; Canfield et al., 2021), phi47 is placed into cluster III, while VPE25 is in cluster X. Together, these genetic analyses demonstrate the lack of common genes between phages VPE25 and phi47, making them genetically distinct.

### 3.2 Phage infection of *E. faecalis* promotes mutations in cell wall macromolecule‐encoding genes necessary for phage infection, and unique mutations accumulate in a phage‐dependent manner

To identify bacterial mutations that confer phage resistance in *E. faecalis* SF28073, we conducted two independent coevolution experiments, infecting five replicate SF28073 cultures with phages derived from individual plaques of either phage VPE25 or phi47, and passaged these cultures for 14 consecutive days. Bacteria‐only controls were established and treated under identical conditions in the absence of phage infection. Genomic DNA from the bacterial populations was sequenced for each replicate at five‐time points (Days 0, 1, 3, 7, and 14). To identify mutations in the SF28073 genome, sequencing reads were mapped to the closed *E. faecalis* SF28073 reference genome generated in this study by a hybrid assembly of Illumina and Oxford Nanopore MinION sequencing reads. The assembled SF28073 genome consists of the chromosome and three endogenous plasmids designated pSF1, pSF2, and pSF3 (GenBank accession numbers CP060804, CP060801, CP060802, and CP060803, respectively).

Nonsynonymous, unique bacterial SNPs were observed in all experimental replicates, except in one of the VPE25‐challenged replicates where the mutation frequencies were below our 30% population‐wide cutoff. Interestingly, the mutations selected in *E. faecalis* SF28073 challenged with phage VPE25 largely differed from mutations in *E. faecalis* SF28073 challenged with phi47. As expected, we observed mutations in *pip*_*EF*_ at one or more time points in all VPE25 challenged replicates, except the culture mentioned above which did not meet our read mapping cutoff (Table A1). We detected *epa* mutations in four of the cultures infected with phi47 (Table A2). These two macromolecules have been previously reported to be essential for successful infection of these phages; the integral membrane protein PIP_EF_ is the receptor for VPE25, while both VPE25 and phi47 rely on the enterococcal polysaccharide antigen (Epa) for adsorption (Chatterjee et al., 2019, 2020; Duerkop et al., 2016).

We identified *ccpA* as the only common gene mutated when SF28073 was challenged with either VPE25 or phi47 (Tables A1 and A2). When exposed to phage VPE25, *ccpA* had mutations in two replicates which appeared at different time points (Days 7 and 14, Table A1), while exposure to phi47 resulted in one replicate harboring a *ccpA* mutation on Day 14 (Table A2). In *E. faecalis*, catabolite control protein A (CcpA) plays a key role in regulating the transcription of proteins involved in carbon source utilization (Gao et al., 2013). Moreover, both experimental groups had mutations arise in different components of the SUF system, which is involved in the iron–sulfur (Fe–S) cluster assembly pathway (Srour et al., 2020) (Tables A1 and A2). One VPE25‐challenged replicate had a mutation in *sufU*, encoding a sulfur relay protein (Srour et al., 2020), while one phi47‐challenged replicate had a mutation in *sufD*. Both mutations were identified on Day 7 and were maintained until Day 14. This suggests that both phages may utilize bacterial iron–sulfur complexes during infection. We also found a mutation in the gene *manX* which was identified in one replicate challenged with VPE25. *manX* is a component of the *manXYZ* operon encoding a protein complex involved in the transportation of mannose as well as other carbohydrates (Plumbridge, 1998).

Additionally, one replicate challenged with phage VPE25 had mutations in genes encoding a putative restriction‐modification (R–M) system, located within the two specificity (S) subunit genes, specifically genes H9Q64_13860, and H9Q64_13845 (Table A1). In R–M systems, the S subunit genes, composed of two target recognition domains, recognize specific DNA sequences thereby providing target specificity to the R–M complex (Gao et al., 2011). On Day 7, both subunits shared missense mutations resulting in amino acid changes from leucine to phenylalanine and lysine to glutamine. Surprisingly, on Day 14 these mutations were no longer detected, suggesting that they rendered these cells less fit in the population. Other nonsynonymous mutations specific to each S subunit gene were also found on Day 7. These two mutations were observed again on Day 14 at higher frequencies, which on the contrary, suggest that these mutations provided a fitness advantage to the population. Lastly, Day 14 revealed two additional mutations in both S subunit‐encoding genes that were not present on Day 7. The numerous amino acid changes observed across the S subunits of the R–M system suggest these mutations may be increasing the specificity of the subunit S towards recognizing the VPE25 genome.

When *E. faecalis* SF28073 coevolved with phi47, we observed three genes mutated across multiple replicates (Table A2). H9Q64_01755, a predicted transposase, was mutated in two replicates. Both replicates had the same mutation resulting in a change from arginine 144 to leucine. H9Q64_09795 was also mutated in two replicates. This gene, *epaAC*, is a predicted epimerase/dehydratase (Guerardel et al., 2020). Lastly, H9Q64_09850 was mutated in three replicates. This gene is *epaR*, the final gene in the rhamnose‐sugar biosynthesis locus of *epa* that is a predicted priming glycosyltransferase (Guerardel et al., 2020). Epa is involved in phage adsorption (Chatterjee et al., 2019; Ho et al., 2018), making this gene essential for successful phi47 infection.

**Figure 2:**
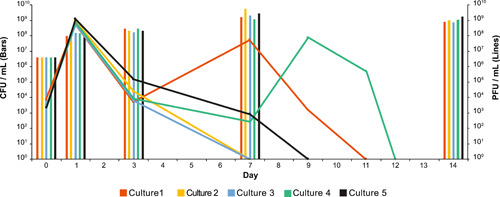
Phi47 kinetics differ in each experimental replicate. Bars represent the CFU/ml shown on the left *Y*‐axis, lines represent the PFU/ml shown on the right *Y*‐axis. Lines and bars of the same color represent *E. faecalis* SF28073 and phi47 titers, respectively. Bars represent the mean of three technical replicates, while lines are a single biological replicate. Additional plaque assays were performed to identify when phi47 became undetectable in each culture

We looked closer at the distribution of these mutations among single clones to better understand their distribution in the population. We sequenced a total of seven colonies, all of which are resistant to phi47, corresponding to Day 7 Cultures 1, 4, and 5 (Table A3). We selected Day 7 since it was the last sample where the bacteria were cocultured with phi47, and these cultures were selected due to their different phi47 kinetics on Day 7 (Figure 2). We observed *epa* mutations in all seven colonies, including those derived from Culture 4, indicating that the frequency of *epa* mutations in Culture 4 was below our limit of detection by whole‐genome sequencing but can be resolved when analyzing single clones. All of the isolates from Culture 1 shared mutations in *ptsP, sufD*, and a transposase.

**Figure 3:**
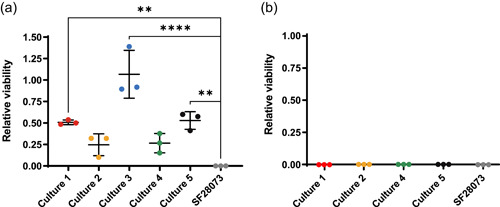
Resistance to phi47 protects bacteria from VPE25 infection. Serial dilutions of Day 7 bacterial communities were challenged with the opposite phage that the bacteria was coevolved with. (a) *E. faecalis* SF28073 that was coevolved with phi47 was challenged with VPE25. (b) *E. faecalis* SF28073 that was coevolved with VPE25 was challenged with phi47. Multiple comparisons using a one‐way ANOVA. **p ≤ 0.01, ****p ≤ 0.0001

To determine if the mutations protected SF28073 from infection by other phages, we challenged bacteria from Day 7 cultures with the phage it was not challenged with (Figure 3).

Bacteria from Day 7 cultures were grown overnight, normalized to an OD of 0.1, and spotted on agar plates that contain phage, or agar without phage. Bacteria that were coevolved with phi47 are resistant to infection by VPE25 (Figure 3a), while bacteria that were coevolved with VPE25 are susceptible to phi47 infection (Figure 3b).

### 3.3 Phi47 acquires mutations in tail and hypothetical genes during coevolution with *E. faecalis* SF28073

Phi47 is an uncharacterized phage; little is known about genes required for phi47 infection other than its dependence on Epa (Chatterjee et al., 2019). To further explore the mechanisms of phi47 infection, we enumerated both phi47 and *E. faecalis* SF28073 to determine the population kinetics for each experimental replicate (Figure 2) and sequenced the phi47 population throughout our experiment. We observed different phage abundance patterns across the five replicates, despite each being treated identically. While all replicates had an expected spike in phi47 titer on Day 1 after 24 h of replication in a completely susceptible population, and a reduction in titer on Day 3, phage abundance differed for each replicate on Day 7 (Figure 2). Culture 1 phi47 titer spiked and was followed by a continuous decline until phi47 was no longer detectable in the culture via plaque assay by Day 11. Cultures 2 and 3 had no detectable phi47 on Day 7. Cultures 4 and 5 had low phage titers on Day 7. Culture 4 had a phi47 spike on Day 9 followed by a decline until it was no longer detected on Day 12, and phi47 was undetectable in Culture 5 by Day 9.

Due to the differences observed in phi47 abundance throughout these experiments, we sequenced the phage population of each replicate on Days 0, 1, 3, and 7. Day 14 was excluded from analysis because no replicate had detectable phages by plaque assay. Sequencing of phage DNA revealed that each culture had a unique mutation profile. Table 3 in the Appendix (Table A3) includes all observed mutations, including those in intergenic regions and synonymous changes. In our analysis, we were most interested in SNPs that result in nonsynonymous changes. Phages from Cultures 2 and 3 acquired the most SNPs, but viable phages were undetectable at Day 7, suggesting that these acquired mutations in the various tail and hypothetical protein‐encoding genes may have been deleterious to the phages' ability to overcome bacterial resistance mutations. Phages from Cultures 3 and 4 developed identical tail SNPs on Day 7. While there were no infectious phages detectable by plaque assay in Culture 3 on Day 7, we were still able to recover phage DNA in culture media, allowing us to perform genetic analyses. Culture 5 phages only developed one SNP in the gene encoding the tail fiber protein on Day 3, which was maintained on Day 7. Culture 1 phages developed no SNPs. Interestingly, there are three hypothetical genes, the major tail protein gene, and one tRNA that were mutated in phages across multiple cultures (Table A4). Despite the different mutations observed across replicates, phi47 was not detectable in any of the cultures by the end of the passaging, indicating that these phages were unable to subvert phage resistance leading to the inability to detect them by our methods.

## 4 DISCUSSION

*E. faecalis* is a commensal and nosocomial pathogen and is becoming increasingly resistant to last‐resort antibiotics (Turner, Lee, et al., 2021). In this study, we show the coevolution of *E. faecalis* SF28073 with two genetically distinct phages, VPE25 and phi47. Our results reveal that each phage selects for different *E. faecalis* mutations, indicating that genetically unique phages select different genetic compositions within the population. In particular, for both phages, *E. faecalis* developed missense mutations in genes encoding cell wall macromolecules, specifically PIP_EF_ and Epa, that are required for successful infection by VPE25 and phi47, respectively (Chatterjee et al., 2019; Duerkop et al., 2016). VPE25 has recently been shown to depend on Epa (Chatterjee et al., 2020), most likely for adsorption to the cells. Because PIP_EF_ and Epa are essential for successful phage infection, mutations in these genes prevent phage infection. Despite this, we observed no *epa* mutations in cultures challenged with VPE25, suggesting that *pip*_*EF*_ mutations are dominant and potentially more advantageous than *epa* mutations, likely due to fitness costs (Chatterjee et al., 2019; Ho et al., 2018). SNPs in *pip*_EF_ and *epa* indicate that phage receptor and coreceptor mutations are common to prevent phage infection. Additionally, many enterococcal phages rely on Epa for adsorption to cells (Al‐Zubidi et al., 2019; Canfield et al., 2021; Chatterjee et al., 2019, 2020; Ho et al., 2018). In this study, we show that *epa* mutations are abundant when the bacteria are challenged with phi47. Mutations in *epa* genes also protect bacteria against VPE25 infection (Figure 3a). Therefore, if phi47 were to be used in a therapeutic cocktail with other Epa‐dependent enterococcal phages, *epa* mutations could reduce the effectiveness of the cocktail. If the bacteria develop dominant mutations in alternative phage receptors, such as *pip*_*EF*_, Epa will remain intact. This could promote infection by Epa‐dependent phages that utilize receptors other than *pip*_*EF*_ (Figure 3b). Our data suggest that VPE25 and phi47 could be useful in combination as a therapeutic cocktail, yet there remains a need to identify Epa‐independent phages for inclusion in future therapeutic phage cocktails.

Mutations in *ccpA* were found in cultures challenged with both VPE25 and phi47. CcpA, a transcriptional regulator, plays a central role in the catabolite control mechanism, regulating transcription in response to carbon source availability (Carvalho et al., 2011; Gao et al., 2013; Muscariello et al., 2001). Phage infection and development are determined by bacterial host factors such as nutritional state (Li et al., 2003). A recent study showed that carbon source is an important factor in phage production (Silva et al., 2021). A previous study using *Bacillus subtilis* showed that phage 29 infection downregulated CcpA‐dependent genes. The authors propose that repression of genes involved in the utilization of specific carbon sources keeps *B. subtilis* in a metabolic state ideal for phage development (Li et al., 2003), suggesting a link between CcpA and optimal phage production. It is possible that nonsynonymous mutations in *ccpA* alter carbohydrate metabolism. Studies focused on transcription during bacteria and phage coevolution could reveal deeper insights into the impact of *ccpA* mutation. Studies in streptococci have established a role for CcpA in the control of virulence factors and carbohydrate metabolism (Carvalho et al., 2011). We cannot rule out the possibility that CcpA may be directly involved in the regulation of *epa* transcription or indirectly involved by regulating genes responsible for the production of Epa precursors. Either circumstance could influence the production of Epa thereby affecting phage infection.

A mutation in the gene *manX*, a component of the *manXYZ* operon, was found in one replicate infected with VPE25. The ManXYZ protein complex is a transporter for mannose as well as other molecules, such as *N*‐acetylglucosamine (GlcNAc) (Plumbridge, 1998). Interestingly, analysis of Epa from two *E. faecalis* strains revealed the presence of GlcNac (Guerardel et al., 2020). In addition, a coevolution study using phage ΦSA012 and its host, *Staphylococcus aureus* SA003, demonstrated that the phage receptor‐binding protein binds to α‐GlcNAc in wall teichoic acids (Takeuchi et al., 2016). Combined, these data suggest that mutations in the *manX* gene could have an impact on the production of Epa or other exopolysaccharides involved in phage adsorption, likely affecting phage infection.

We also show that phi47 develops mutations during coculture with its host. Phages from Cultures 3 and 4 developed identical mutations in the gene encoding the major tail protein on Day 7. However, Culture 3 had no phages detectable by plaque assay on that day, suggesting that these phages were unable to overcome the bacterial mutations in cell wall‐associated genes, such as *epa*. Phages in Culture 4 developed the same major tail protein mutations before the bacteria developed *epa* mutations, suggesting that the major tail protein mutations arose independently of host *epa* mutations. Despite these novel mutations, phi47 could not be maintained in the experimental cultures.

While there is currently only one paper investigating phage and *Enterococcus* coevolution (Wandro et al., 2019), numerous coevolution studies have been performed with other Gram‐positive bacteria and their phages (Peters et al., 2020; Takeuchi et al., 2016) and the model organism *E. coli* and its phages. For example, in one study *E. coli* and phage T3 were coevolved in a chemostat, allowing for a controlled experimental environment. Under this condition, the authors observed common bacterial mutations at the gene level and phage mutations at the codon level across experimental replicates (Perry et al., 2015). We believe that our current study both supports and contradicts these findings. In our study, four of the five cultures challenged with phage VPE25 developed mutations in *pip*_EF_. This maintains the conclusion that bacterial mutations in response to phage pressure reproducibly occur at the gene level. However, cultures challenged with phi47 showed more variability among *E. faecalis* genomic mutations across replicates. While phi47 developed some mutations that were shared across multiple cultures, each culture had a unique phage SNP profile. This outcome may depend on the phage–host bacterial pair used in coevolution experiments, the specific MOI used to initially infect the cultures, or the growth conditions tested.

Lack of reproducibility in coevolution experiments may be due to abiotic selection pressures, meaning that divergence among experimental replicates can increase via random events occurring in each population over time. This argues for a more controlled and consistent experimental design, such as a chemostat for continuous culturing, that may reduce stochastic events. We believe that our experimental design, which included manual daily subculturing, may have introduced bottlenecks causing a selection bias for the growth and preservation of bacteria while causing phages to fall below our detection limit. In our experiments, we inoculated each flask with ~10^8^ CFU of *E. faecalis* SF28073 and ~10^5^ PFU of phage (MOI 0.001). A similar ratio (MOI 0.003) was used to study phage‐*E. faecium* coevolution (Perry et al., 2015). However, for the *E. faecium* study, passaging was performed at a 1:10 ratio, transferring every 12 h for a total of 16 times, while we implemented a passage ratio of 1:100, transferring every 24 h for 14 days.

A study in *E. coil* used a chemostat with an MOI of 2, showing that in this setting coevolution happened in the form of adaptation and counter‐adaptation (Mizoguchi et al., 2003). While we began to observe phage extinction by Day 7, the phage population in the above‐mentioned *E. faecium* study was maintained in all cultures throughout the experiment. We speculate that the small volume we subcultured, the low starting MOI, and the fact that there were magnitudes more bacterial cells than phage in the population, may have caused a significant decrease in the number of phages passaged, thus introducing a bottleneck that ultimately eliminated the phage from the population.

Our study highlights the importance of considering experimental design such as MOI and subculturing methods when studying the coevolution of phages and their hosts to prevent the introduction of bottlenecks. Similar to studies using transposon‐insertion libraries that can be confounded by the presence of bottlenecks (Abel et al., 2015), bottlenecks could prevent the discovery of novel genes involved in phage infection by limiting the presence of the phages in the population. Future studies should consider our methods and modify them to support continuous phage replication—for instance, using higher volumes if manually passaging, implementing a higher starting MOI, and the use of continuous culturing systems.

## CONFLICTS OF INTEREST

The authors declare no conflicts of interest.

## ETHICS STATEMENT

None required.

## AUTHOR CONTRIBUTIONS

**Cydney N. Johnson**: conceptualization (equal), data curation (equal), formal analysis (equal), funding acquisition (equal), investigation (equal), methodology (equal), supervision (equal), validation (equal), visualization (equal), writing—original draft (equal), writing—review & editing (equal). **Dennise P. Araya**: conceptualization (equal), data curation (equal), formal analysis (equal), investigation (equal), methodology (equal), supervision (equal), validation (equal), visualization (equal), writing—original draft (equal), writing—review & editing (equal). **Viviane Schink**: data curation (supporting), formal analysis (supporting), investigation (supporting), methodology (supporting). **Moutusee Islam**:  data curation (supporting), formal analysis (supporting), methodology (supporting). **Mihnea R. Mangalea**: data curation (supporting), formal analysis (supporting), funding acquisition (equal), methodology (supporting), writing—review & editing (supporting). **Emily Decurtis**: data curation (supporting), formal analysis (supporting), investigation (supporting). **Tuong‐Vi C. Ngo**:  data curation (supporting), formal analysis (supporting), investigation (supporting). **Kelli Palmer**: conceptualization (equal), data curation (equal), formal analysis (equal), funding acquisition (equal), methodology (equal), project administration (equal), resources (equal), supervision (equal), writing—review & editing (equal). **Breck Duerkop**: conceptualization (equal), data curation (equal), formal analysis (equal), funding acquisition (equal), methodology (equal), project administration (equal), resources (equal), supervision (equal), writing—review & editing (equal).

## Data Availability

All data are provided in full in this paper, except for raw Illumina DNA sequencing reads. Sequencing reads are available at the European Nucleotide Archive (ENA) under project accession PRJEB48380: http://www.ebi.ac.uk/ena/browser/view/PRJEB48380

## Appendix

See Tables A1, A2, A3, and A4

**Table A1:** Mutations present in *E. faecalis* SF28073 challenged with VPE25

Culture	Position	Gene	Predicted function	AA Change	Frequency (%)
*Day 1*					
1	1081166	H9Q64_06285	Phage Infection Protein (PIP)	Ser36*	99
2	1081166	H9Q64_06285	Phage Infection Protein (PIP)	Ser36*	96
4	1081166	H9Q64_06285	Phage Infection Protein (PIP)	Ser36*	87
	2429489	Intergenic region	Downstream of H9Q64_13025	NA	94
*Day 3*					
2	1079889	H9Q64_06285	Phage Infection Protein (PIP)	Gln462*	33
	2914320	H9Q64_15390	PTS mannose/fructose/sorbose transporter family subunit IID	Asn88fs	33
*Day 7*					
1	2912438	*manX*	ManX	Asn77Lys	49
	1081166	H9Q64_06285	Phage Infection Protein (PIP)	Ser36*	99
2	2576910, 2576915, 2576930	H9Q64_13860	Restriction endonuclease subunit S	Leu166Phe, Glu165Lys, Thr160Ala	30, 39, 40
	2573919, 2573916, 2573901	H9Q64_13845	Restriction endonuclease subunit S	Leu181Phe, Lys180Glu, Ala175Thr	32, 37, 39
4	1079220	H9Q64_06285	Phage Infection Protein (PIP)	Ser685fs	56
	2520234	Intergenic region	Downstream of H9Q64_13605	NA	30
	240029	*ccpA*	Catabolite control protein A	Asn27Ile	58
5	1079058	H9Q64_06285	Phage Infection Protein (PIP)	Ile739fs	39
	1963417	*sufU*	SufU, component of the SUF system	Thr70Ile	40
*Day 14*					
1	240229	*ccpA*	Catabolite control protein A	Tyr94Asn	33
	2912438	*manX*	ManX	Asn77Lys	43
	1858079	Intergenic region	Upstream of H9Q64_10120	NA	37
	1081166	H9Q64_06285	Phage Infection Protein (PIP)	Ser36*	96
2	2576939, 2576930	H9Q64_13860	Restriction endonuclease subunit S	Asn157Asp, Thr160Ala	47, 56
	2573891, 2573901	H9Q64_13845	Restriction endonuclease subunit S	Asp172Asn, Ala175Thr	43, 83
5	1079058	H9Q64_06285	Phage Infection Protein (PIP)	Ile739fs	71
	1157154	Intergenic region	Upstream of H9Q64_06630	NA	46
	1963417	*sufU*	SufU, component of the SUF system	Thr70Ile	72

*Note*: Mutations found in bacterial controls are excluded from this final list. NA indicates no change in amino acid. The *indicate a codon change to a stop codon.

**Table A2:** Mutations present in *E. faecalis* SF28073 challenged with phi47

Culture	Position	Gene	Predicted function	AA change	Frequency (%)
*Day 1*					
None detected					
*Day 3*					
4	127840	H9Q64_RS01755	Transposase	Arg144Leu	97
*Day 7*					
1	1233173	*ptsP*	Phosphoenolpyruvate‐protein phosphotransferase	Asp267Ala	70
	1432264	Intergenic region	Upstream of H9Q64_RS07940	NA	33
	1779309	H9Q64_RS09795	Epimerase, *epaAC*	Glu208*	66
	1965247	*sufD*	FeS assembly protein	Arg295Gln	66
	2429489	H9Q64_RS13020	16S ribosomal RNA	NA	93
2	1779359	H9Q64_RS09795	Epimerase, *epaAC*	Gly191Asp	31
4	127840	H9Q64_RS01755	Transposase	Arg144Leu	97
*Day 14*					
1	240867	*ccpA*	Catabolite control protein A, transcription negative regulator	Leu306Phe	34
	1233173	*ptsP*	Phosphoenolpyruvate‐protein phosphotransferase	Asp267Ala	32
	1451382	H9Q64_RS08040	DUF1189 domain‐containing protein	Ala133Thr	42
	1779309	H9Q64_RS09795	Epimerase, *epaAC*	Glu208*	36
	1793154	H9Q64_RS09850	Sugar transferase, *epaR*	Pro320Leu	43
	1965247	*sufD*	FeS assembly protein	Arg295Gln	31
2	1779359	H9Q64_RS09795	Epimerase, *epaAC*	Gly191Asp	43
3	1793226	H9Q64_RS09850	Sugar transferase, *epaR*	Thr296Ile	43
4	1157622	Intergenic region		NA	39
5	1234192	H9Q64_RS06955	Directly upstream of ptsP, phosphocarrier protein HPr	Ala16Val	44
	1793154	H9Q64_RS09850	Sugar transferase, *epaR*	Pro320Leu	52

*Note*: Mutations found in bacterial controls are excluded from this final list. NA indicates no change in amino acid. The *indicate a codon change to a stop codon.

**Table A3:** Mutations present in single phage‐resistant colonies of *E. faecalis* SF28073 challenged with phi47

Isolate	Position	Overlapping annotations	Predicted function	Amino acid change	Frequency (%)
Culture 1 Colony 1	127840	H9Q64_RS01755	Transposase	Arg144Leu	97
	1233173	*ptsP*	Phosphoenolpyruvate‐protein phosphotransferase	Asp267Ala	100
	1284755	H9Q64_RS07195	MATE family efflux transporter	NA	100
	1779309	H9Q64_RS09795	Epimerase, *epaAC*	Glu208*	100
	1965247	*sufD*	FeS assembly protein	Arg295Gln	100
Culture 1 Colony 2	127840	H9Q64_RS01755	Transposase	Arg144Leu	99
	1233173	*ptsP*	Phosphoenolpyruvate‐protein phosphotransferase	Asp267Ala	99
	1779309	H9Q64_RS09795	Epimerase, *epaAC*	Glu208*	99
	1965247	*sufD*	FeS assembly protein	Arg295Gln	99
Culture 1 Colony 3	127840	H9Q64_RS01755	Transposase	Arg144Leu	98
	1233173	*ptsP*	Phosphoenolpyruvate‐protein phosphotransferase	Asp267Ala	100
	1284755	H9Q64_RS07195	MATE family efflux transporter	NA	100
	1779309	H9Q64_RS09795	Epimerase, *epaAC*	Glu208*	100
	1965247	*sufD*	FeS assembly protein	Arg295Gln	99
	2429489	H9Q64_RS13020	16S ribosomal RNA	NA	96
Culture 4 Colony 1	1779485	H9Q64_RS09795	Epimerase, *epaAC*	Ala149Asp	99
	2909682	H9Q64_RS15370	Sigma 54‐dependent transcriptional regulator	Ala439insValLys	82
Culture 5 Colony 1	127840	H9Q64_RS01755	Transposase	Arg144Leu	33
	1234192	H9Q64_RS06955	Directly upstream of ptsP, phosphocarrier protein HPr	Ala16Val	100
	1793154	H9Q64_RS09850	Sugar transferase, *epaR*	Pro320Leu	100
Culture 5 Colony 2	1488410	H9Q64_RS08225	PTS mannitol transporter subunit IICBA	Met1?	99
	1792160	H9Q64_RS09845	Glucosyltransferase, *epaS*	Gln141*	100
Culture 5 Colony 3	1779552	H9Q64_RS09795	Epimerase, *epaAC*	Val127Phe	99
	2910925	H9Q64_RS15370	Sigma 54‐dependent transcriptional regulator	Ile854fs	99

*Note*: Mutations found in bacterial controls are excluded from this final list. NA indicates no change in amino acid. The *indicate a codon change to a stop codon.

**Table A4:** Mutations present in phi47 coevolved with *E. faecalis* SF28073

Culture	Position	Reference	Allele	Annotation	AA change	Frequency (%)
*Day 1*						
None detected						
*Day 3*						
2	2678	T	G	Upstream of tail fiber protein	NA	100
	7802	‐	T	Minor capsid protein	Thr594fs	100
	15447	T	A	Hypothetical protein	NA	98
	15450	A	C		NA	98
	21557	A	T	Hypothetical protein	NA	100
	21559	A	T		Glu112Val	100
	21562	G	T		Arg113Leu	100
	27538	C	G	Hypothetical protein	Glu4Gln	31
	29858	T	A	Upstream of hypothetical protein	NA	100
	29860	T	A	Upstream of hypothetical protein	NA	100
	29876	G	A	Hypothetical protein	Pro122Leu	100
	31004	C	‐	Hypothetical protein^a^	Val14fs	39
	31009	‐	A		Lys12fs	56
	31285	C	G	Hypothetical protein	Arg51Thr	99
	33015	T	A	Hypothetical protein	Lys88Ile	100
	34228	C	‐	Hypothetical protein^a^	Gly59fs	52
	35922	A	‐	Hypothetical protein	Ser94fs	100
	41444	T	‐	Hypothetical protein	Asn59fs	91
	44217	‐	A	RuvC‐like crossover junction endodeoxyribonuclease	Gln54fs	93
	45366	C	G	Hypothetical protein^a^	Glu126Asp	100
	45368	C	G		Glu126Gln	100
	45373	A	T		Met124Lys	100
	46212	C	A	Upstream of HNH endonuclease	NA	100
	50818	C	T	Hypothetical protein^a^	NA	92
	52148	GG	TA	tRNA‐Pseudo‐TGA^a^	NA	100
	52155	C	T		NA	100
	52164	CCAA	TTGC		NA	100
	52171	C	G		NA	100
	52178	A	C		NA	100
	52184	AT	GA		NA	100
	55247	‐	C	Hypothetical protein	Arg63fs	94
3	11561	AA	G	Upstream of phage tail protein	NA	69
	31004	C	‐	Hypothetical protein^a^	Val14fs	63
	31009	‐	A		Lys12fs	37
	31040	T	‐		Met2fs	50
	31057	T	G	Upstream of hypothetical protein	NA	89
	31060	C	A	Upstream of hypothetical protein	NA	89
	48552	A	‐	DNA replication protein	Asn108fs	69
	48556	‐	G		Lys106fs	69
	50818	C	T	Hypothetical protein^a^	NA	100
	52155	C	T	tRNA‐Pseudo‐TGA^a^	NA	100
	52164	CCAA	TTGC		NA	100
	52171	C	G		NA	100
	52178	A	C		NA	100
	52184	AT	GA		NA	96
5	100	A	C	Tail fiber protein	Val859Gly	95
*Day 7*						
2	27651	‐	TG	Hypothetical protein	Asp48fs	67
	27651	C	T		NA	33
	27652	‐	T		Asp48fs	67
	27655	T	C		Asn46Ser	67
	27657	C	A		Met45Ile	67
	31004	C	‐	Hypothetical protein^a^	Val14fs	44
	31009	‐	A		Lys12fs	53
	31040	T	‐		Met2fs	53
	52171	C	G	tRNA‐Pseudo‐TGA^a^	NA	95
	52178	A	C		NA	96
	52184	AT	GA		NA	96
3	10451	C	T	Major tail protein^a^	Val217Ile	100
	10453	G	A		Ala216Val	100
	10457	‐	C		Thr215fs	100
	10459	T	C		Asn214Ser	100
	10463	C	‐		Val213fs	100
	28104	C	T	Hypothetical protein	Glu4Lys	100
	28107	G	A		Pro3Ser	100
	28114	GG	‐	Upstream of hypothetical protein	NA	100
	31004	C	‐	Hypothetical protein^a^	Val14fs	36
	31009	‐	A		Lys12fs	64
	31040	T	‐		Met2fs	56
	31057	T	G	Upstream of hypothetical protein	NA	89
	31060	C	A	Upstream of hypothetical protein	NA	88
	34228	C	‐	Hypothetical protein^a^	NA	
	45366	C	G	Hypothetical protein^a^	Glu126Asp	100
	45368	C	G		Glu126Gln	100
	45373	A	T		Met124Lys	100
	48167	G	‐	DNA replication protein	Arg236fs	100
	48184	T	C		Glu230Gly	100
	50818	C	T	Hypothetical protein	NA	100
	52155	C	T	tRNA‐Pseudo‐TGA^a^	NA	100
	52164	CCAA	TTGC		NA	100
	52171	C	G		NA	100
	52178	A	C		NA	100
	52184	AT	GA		NA	100
4	10451	C	T	Major tail protein^a^	Val217Ile	38
	10453	G	A		Ala216Val	38
	10457	‐	C		Thr215fs	35
	10459	T	C		Asn214Ser	35
	10463	C	‐		Val213fs	33
	17578	‐	C	Phage terminase, large subunit	Tyr358fs	50
	52171	C	G	tRNA‐Pseudo‐TGA^a^	NA	37
	52178	A	C		NA	37
	52184	AT	GA		NA	34
5	100	A	C	Tail fiber protein	Val859Gly	93
	31516	‐	C	Upstream of hypothetical protein	NA	35
	31520	TT	AA	Upstream of hypothetical protein	NA	35

*Note*: NA indicates no change in amino acid.

^a^ Genes mutated across multiple cultures.
